# Using a Positive Deviance Approach to Influence the Culture of Patient Safety Related to Infection Prevention

**DOI:** 10.1093/ofid/ofy231

**Published:** 2018-10-01

**Authors:** Pranavi Sreeramoju, Lucia Dura, Maria E Fernandez, Abu Minhajuddin, Kristina Simacek, Thomas B Fomby, Bradley N Doebbeling

**Affiliations:** 1 Division of Medicine–Infectious Diseases, UT Southwestern Medical Center, Dallas, Texas; 2 Department of Infection Prevention, Parkland Health and Hospital System, Dallas, Texas; 3 Department of English, Rhetoric & Writing Studies, The University of Texas at El Paso, El Paso, Texas; 4 Department of Clinical Sciences, UT Southwestern Medical Center, Dallas, Texas; 5 Department of Sociology, Indiana University, Bloomington, Indiana; 6 Department of Economics, Southern Methodist University, Dallas, Texas; 7 Department of Biomedical Informatics, College of Health Solutions, Arizona State University, Phoenix, Arizona

**Keywords:** positive deviance, infection control, health care personnel, patient safety culture, social networks

## Abstract

**Background:**

Health care–associated infections (HAIs) are a socio-technical problem. We evaluated the impact of a social change intervention on health care personnel (HCP), called “positive deviance” (PD), on patient safety culture related to infection prevention among HCP.

**Methods:**

This observational study was done in 6 medical wards at an 800-bed public academic hospital in the United States. Three of these wards were randomly assigned to receive PD intervention on HCP. After a retrospective 6-month baseline period, PD was implemented over 9 months, followed by 9 months of follow-up. Patient safety culture and social networks among HCP were surveyed at 6, 15, and 24 months. Rates of HAI were measured among patients.

**Results:**

The measured patient safety culture was steady over time at 69% aggregate percent positive responses in wards with PD vs decline from 79% to 75% in wards without PD (F statistic 10.55; *P* = .005). Social network maps suggested that nurses, charge nurses, medical assistants, ward managers, and ward clerks play a key role in preventing infections. Fitted time series of monthly HAI rates showed a decrease from 4.8 to 2.8 per 1000 patient-days (95% confidence interval [CI], 2.1 to 3.5) in wards without PD, and 5.0 to 2.1 per 1000 patient-days (95% CI, –0.4 to 4.5) in wards with PD.

**Conclusions:**

A positive deviance approach appeared to have a significant impact on patient safety culture among HCP who received the intervention. Social network analysis identified HCP who are likely to help disseminate infection prevention information. Systemwide interventions independent of PD resulted in HAI reduction in both intervention and control wards.

Health care–associated infections (HAIs) continue to be a significant public health problem [1, 2] despite advances in tools and strategies available for reducing them. An explanation for why HAIs continue to occur may be that they are a socio-technical problem. Technical strategies to reduce HAIs, such as making hand sanitizer and hand washing sinks available and establishing protocols for environmental cleaning and safe insertion of catheters, may need an addition of adaptive strategies [3]. Strategies that address social and behavioral norms among health care personnel related to their use of infection prevention measures, collectively referred to as “patient safety culture,” have not been studied well. Positive deviance (PD) [4–8] is a strategy that has gained attention in recent years. It was previously used to successfully solve seemingly intractable and complex social and public health problems. Through intentional inquiry, the PD approach explores the social aspects of infection prevention practices among health care personnel. In addition to identifying barriers and potential solutions, the approach focuses on identifying and deploying peer role models to generate positive peer pressure and mobilize change.

PD has been used to reduce infections caused by methicillin-resistant *Staphylococcus aureus* in Veterans Affairs hospitals [9], improve hand hygiene [10, 11], and reduce surgical site infections [12]. However, these studies did not explore the impact on patient safety culture among health care personnel (HCP), especially in the setting of parallel system-wide horizontal infection prevention interventions occurring independent of PD strategy. They also did not discuss the methodology of implementing PD in sufficient detail. In this study, we evaluate the impact of PD on the patient safety culture related to infection prevention among HCP. Here we describe our implementation methodology so that future studies may replicate and refine the approach as needed. Improvements in patient safety culture among health care personnel are necessary for sustainment of HAI reduction.

## METHODS

The study was done at Parkland Memorial Hospital, an 800-bed public academic medical center in Dallas, Texas, from April 2011 to March 2013. We conducted an observational prospective study with a retrospective baseline period in 6 wards. Three of these wards were randomly selected for health care personnel to receive positive deviance intervention. The baseline period was April 2011 to September 2011. PD was implemented from October 2011 to June 2012. The follow-up period was from July 2012 to March 2013. The wards serve patients with general medical problems, in addition to those needing specialty care like geriatrics, hematology/oncology, and dialysis care. Wards in the intervention group were not significantly different in patient volume compared with the control wards. Other attributes of the wards like HAI rates and culture of safety were unknown at the time of random allocation of the intervention. All employed HCP (nurses, patient care technicians, ward managers, and clerks) and all patients receiving care in the study wards were included. HCP who do not provide care exclusively in a study ward, for example, physicians and respiratory therapists, were excluded to prevent crossover between the intervention and control wards.

### Implementation of Positive Deviance Intervention

The intervention was implemented over 9 months using a semistructured design in a series of 3 steps, where experience with each step informed the next step. The decision on 9 months was based on convenience, as there are no firm timelines for a relatively novel intervention like PD. The steps of the intervention are outlined in Box 1. In the first step, the chief medical officer and chief nursing officer invited HCP to participate, with assurance of voluntariness, freedom from negative consequences for thoughts and ideas expressed, and anonymity, as desired. In the second step, the research staff (P.S., M.E.F., and L.D.) conducted a positive deviance inquiry among HCP. The inquiry was conducted in staff break rooms and nursing stations. The sessions were evenly distributed between the day and night shifts. Drop boxes and graffiti boards were also provided for HCP to offer thoughts and ideas anonymously. The line list of patients with HAI and unit level rates of HAI were discussed with HCP willing to engage with the research team. During the PD inquiry, the HCP identified 12 “positive deviants” among themselves who are “role models” for infection prevention practices in the wards where they provide care. In the third step, which occurred at 6 months from start of intervention, the information gathered by the study team was collated by an action planning group that consisted of the “positive deviants,” managers of the 3 wards, 2 infection preventionists, and a research team member (M.E.F.). The group organized and prioritized the large number of ideas and made plans to disseminate and implement them. Most importantly, they owned their plans, which included making patients their partners in preventing infections and wanting to improve their own infection prevention skills. This third step lasted the remaining 4 months of the intervention period. All activities and time spent on the intervention were documented. During the follow-up period, the research staff ceased involvement in the intervention wards.

**Box 1. Steps of Positive Deviance Intervention**

*Step 1*: Leader extends invitation to health care personnel to participate, with assurances that participation in the intervention is voluntary, that their responses will not be judged in any way, that there will be no negative consequences, that they are free to speak their minds, and that they are free to leave the conversation at any time.

*Step 2*: Positive deviance inquiry or “Discovery and Action Dialogues” through 1:1 conversations, focus groups, anonymous drop box reporting, and anonymous graffiti boards in break rooms. The study team asks the following open-ended questions, listens while taking notes, asks any clarifying questions from the participants, and reviews data with the participants if they request. Study team collates responses once the inquiry stops yielding new information.

a. How do you know or recognize when health care–associated infection is present?
b. How do you protect yourself, patients, and others from transmission of any microorganisms?
c. What prevents you from taking these actions all the time?
d. Is there any group or anyone you know who is able to overcome the barriers frequently and effortlessly? How?
e. Do you have any ideas?
f. What initial steps need to be pursued to make it happen? Any volunteers?
g. Who else needs to be involved?

*Step 3*: An action planning group is formed by the “positive deviants” and may include the ward managers and other key personnel. This group owns the organization of ideas and prioritization, implementation, and evaluation of results. The group may implement ideas using Plan-Do-Study-Act cycles, as in traditional quality improvement. Steps 1–3 may be repeated as necessary.

### Outcomes

The outcome of positive deviance intervention was a culture of safety, which was measured using the hospital survey of patient safety climate [13], adapted to infection prevention (see Box 2 for the tool). Social networks, that is, the networks of relationships between HCP and other personnel with different job roles in their wards [14], were also measured to understand the impact of intervention on HCP interactions. The questions asked in the social network survey were: (1) Who do you work with to prevent infections from occurring on this unit? (Collaboration or Current network); (2) List projects and activities you are involved with and the people with whom you work on those projects and activities (Prevention or Project network); (3) From whom have you gotten new ideas or inspiration that helped your infection prevention efforts? (Innovation or Ideas network); (4) Who would you like to work with in the future on preventing infections in your unit? (Potential or Future network). We administered the culture of safety survey and the social network survey in September 2011 (before intervention), June 2012 (after intervention), and March 2013 (end of follow-up period) to the HCP of all 6 study wards via REDCap [15]. To assess the impact of intervention on infection outcomes, the monthly HAI rate—a composite of central line–associated bloodstream infection (CLABSI), *Clostridium difficile* infection (CDI), catheter-associated urinary tract infection (CAUTI), and hospital-acquired pneumonia (HAP) per 1000 patient-days—was measured among patients through review of medical records by the study team and application of surveillance definitions [16] per the Centers for Disease Control and Prevention National Health Safety Network. Composite HAI rate was chosen mainly because the PD inquiry was open-ended and not targeted to any specific HAI. Patients who developed HAI were also evaluated for development of any of the 3 complications during the remainder of their hospital stay, permanent loss of organ or organ system function, transfer to higher level of care, and death. Attributability was not assessed.

**Box 2. Modified Hospital Survey of Patient Safety Climate Adapted to Infection Prevention**

A. **Think about your hospital work area/unit.**
1. People support one another in this unit.
2. When a lot of work needs to be done quickly, we work together as a team to get the work done.
3. In this unit, people treat each other with respect.
4. We are actively doing things to improve/prevent health care–associated infections.
5. Mistakes in preventing infections have led to positive changes here.
6. When one area in this unit gets really busy, others help out.
7. After we make changes to improve infection prevention, we evaluate their effectiveness.
B. **Your Supervisor/Manager**
1. My supervisor/manager says a good word when he/she sees a job done according to established infection prevention procedures.
2. My supervisor/manager seriously considers staff suggestions for improving infection prevention.
3. Whenever pressure builds up, my supervisor wants us to work faster, even if it means taking shortcuts.
4. My supervisor/manager overlooks infection prevention problems that happen over and over.
C. **Communications**
1. Staff will freely speak up if they see something that may negatively affect infection prevention.
2. Staff feel free to question the decisions or actions of those with more authority.
3. Staff are afraid to ask questions when something does not seem right.
D. **Frequency of Events Reported**
1. When a mistake is made, but is *caught and corrected before it causes an infection in a patient*, how often is this reported?
2. When a mistake is made, but has *no potential to cause infection in the patient*, how often is this reported?
3. When a mistake is made that *could cause infection in the patient* but does not, how often is this reported?
E. **Preventing Health Care–Associated Infections Grade**
Please give your work area/unit in this hospital an overall grade on preventing health care–associated infections.
F. **Your Hospital**
1. Hospital units do not coordinate well with each other.
2. There is good cooperation among hospital units that need to work together.
3. It is often unpleasant to work with staff from other hospital units.
4. Hospital units work well together to provide the best care for patients.

The University of Texas Southwestern Institutional Review Board approved the study. Concurrently, during the study, the infection prevention program for the hospital conducted independent surveillance for publicly reportable HAI, as required for acute care hospitals by the state of Texas and nationally by the Centers for Medicare and Medicaid Services (CMS), and pursued aggressive systemwide implementation of hand hygiene because of a “systems improvement agreement” [17, 18] with the CMS.

### Statistical Methods

We compared data at the level of groups, the group of 3 wards where HCP received PD intervention and the group of 3 wards where the HCP did not. We modeled binary outcomes of the culture of safety survey using a logistic regression analysis with group, period, and a group × period interaction term in the model. We analyzed the social network survey data qualitatively and by measuring differences in the average number of job roles of HCP each staff member interacts with to prevent HAI. We mapped social networks using UCINET [19] and NetDraw [20]. For analysis of differences in monthly rates of HAI over time, we used the intervention modeling approach of Box and Tiao [21] with inclusion of 6 months’ lag for detection of intervention effect using the SAS PROC ARIMA procedure. We used 0-inflated Poisson regression to analyze the rates of complication in patients with HAI. We used SAS 9.3 software (SAS Institute Inc., Cary, NC) for all analyses. All tests were 2-tailed, and the critical level of α was set at .05.

## RESULTS

### Positive Deviance Intervention

During PD inquiry, which lasted 5 months, a total of 54 PD inquiry sessions, 13 meetings with positive deviants/peer role models and ward managers, 15 meetings between positive deviance consultant (L.D.) and ward managers, and 6 role-playing sessions were conducted. In total, 110 HCP (77% of HCP in intervention wards) voluntarily engaged with the research team, for a total contact time of 116.5 HCP-hours (including 37 hours spent with the mangers of 3 wards). During the action planning phase, which lasted 4 months, the action planning group sifted through more than 210 ideas and practices suggested by HCP as potential solutions for reducing HAI during PD inquiry. Twelve HCP were identified as positive deviants. The group named their efforts “Stop a Bug, Save a Life.”

### Culture of Safety Related to Infection Prevention

Detailed results are presented in Table 1. The measured culture of safety at baseline was significantly more positive among HCP in the control group compared with the intervention group, although the wards were randomly selected. However, in the subsequent surveys, the culture of safety declined significantly in the control group, which did not have positive deviance intervention, whereas it remained unchanged in the intervention group. The trends in overall culture over the duration of the study period were significantly different between the 2 groups.

**Table 1.** **Results of Hospital Survey of Patient Safety Climate in Health Care Personnel in Intervention and Control Groups Before and After Intervention and At the End of the Follow-up Period**

		Before Intervention, %	End of Intervention, %	End of Follow-up, %	Average Difference Before and After Intervention	Average Difference Between Groups	Difference Between Groups Before and After Intervention
					F Statistic (*P* Value)	F Statistic (*P* Value)	F Statistic (*P* Value)
Participation (% invited)	Control	32.40	81.20	69.60	13.10 (<.001)^a^	33.64 (<.001)^a^	26.65 (<.001)^a^
	Intervention	46.30	50.80	45.80			
Aggregate percent positive	Control	79.30	72.70	75.00	36.19 (<.001)^a^	4.62 (.099)	10.55 (.005)^a^
	Intervention	68.60	70.20	69.30			
Percent positive responses per domain							
A. Unit where HCP works	Control	90.60	83.20	82.50	1.75 (0.19)	0.45 (0.64)	0.09 (0.91)
	Intervention	76.30	75.80	74.60			
B. HCP’s supervisor/ manager	Control	68.80	74.70	77.50	0.00 (0.97)	1.95 (0.14)	0.10 (0.90)
	Intervention	71.50	77.00	74.50			
C. Communication	Control	79.60	62.00	72.80	0.01 (0.93)	0.53 (0.59)	0.07 (0.93)
	Intervention	70.70	69.90	70.90			
D. Frequency of events reported	Control	82.40	73.90	82.60	6.89 (0.009)^a^	1.41 (0.25)	0.55 (0.58)
	Intervention	62.00	66.10	70.20			
E. Overall HAI prevention grade for HCP’s unit	Control	80.60	75.60	74.60	1.24 (0.27)	0.75 (0.47)	0.33 (0.72)
	Intervention	74.00	74.20	63.60			
F. Entire hospital	Control	67.40	58.70	55.30	0.01 (0.91)	0.11 (0.90)	0.33 (0.72)
	Intervention	54.50	56.00	54.80			

Positive response was defined as agree, strongly agree, most of the time, always, very good, and excellent for all questions except the negatively worded questions, for which disagree and strongly disagree are considered positive responses.

Abbreviations: HAI, health care–associated infection; HCP, health care personnel.

^a^Differences between intervention and control groups statistically significant at <.05.

### Social Network Analysis

**Figure 1.**
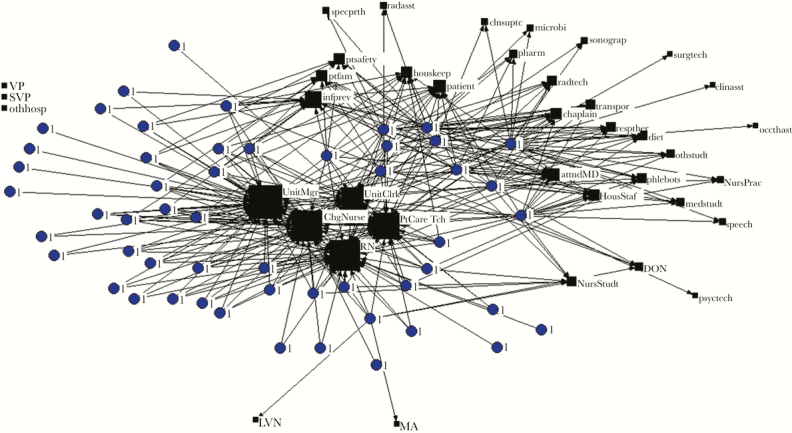
Representative social network map: current network in the intervention group before start of intervention. Round nodes labeled 1 represent health care personnel (HCP) in the intervention group, while square, black nodes represent the job roles of other personnel in the ward they worked with. The size of the nodes is directly proportional to how frequently they worked with someone in that job role. Roles that were not chosen by anyone are not connected to any HCP and are shown in the upper left corner. Abbreviations: attndMD, attending physician; chasst, clinic staff assistant; ChgNurse, charge nurse; chsuptc, clinical support tech; diet, dietician; DON, director of nursing; houskeep, housekeeper; HousStaf, resident physician; infprev, infection prevention; medstudt, medical student; microbi, microbiologist; NursPrac, nurse practitioner; NursStudt, nursing student; occthasst, occupational therapy assistant; othhosp, someone in another hospital; othstudt, other student; pharm, pharmacist; phlebots, phlebotomist; PtCareTch, patient care tech; psyctech, psychiatry technician; ptfam, patient’s family; ptsafety, patient safety; radasst, radiology assistant; radtech, radiology technician; respther, respiratory therapist; RN, registered nurse; specprth, special procedures technician; speech, speech therapist; sonograp, sonographer; surgtech, surgery technician; SVP, senior vice president; transport, transporter; UnitClrk, unitclerk; UnitMgr, unit manager; VP, vice president.

Analysis of social networks revealed that, on average, each HCP worked with 6 to 9 other HCP to prevent infections and reached out to 3 to 5 other HCP for sharing ideas. The number of relationships per HCP did not change significantly in either group during the study period. Social network maps suggested that nurses, charge nurses, patient care technicians, ward managers, and ward clerks play a key role in preventing infections as they are located toward the center of the map, an indicator of high network centrality (representative social network map shown in Figure 1).

### HAI and Complications

**Figure 2.**
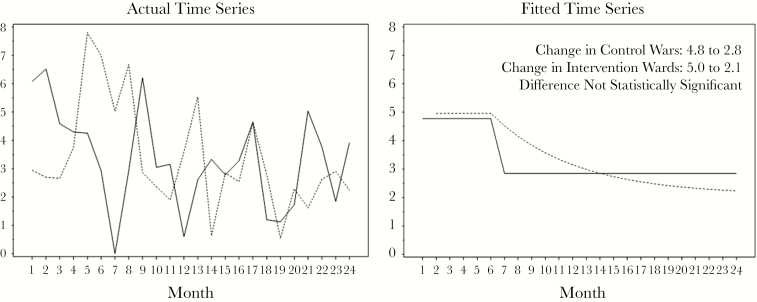
Rates of health care–associated infections (HAIs) in intervention and control groups. Solid line: HAI rate in control wards. Dashed line: HAI rate in wards in which health care personnel received intervention. Y-axis: Rate of health care–associated infections per 1000 patient-days.

During the study period of 2 years, the 6 wards provided care for 11 069 unique patients during 16 876 encounters. Two hundred thirty patients developed 255 HAI: 48 (18.8%) CLABSI, 53 (20.8%) CAUTI, 79 (31%) HAP, and 75 (29.4%) CDI. Actual monthly HAI rates fluctuated widely in both the intervention and control groups of wards. The patterns of HAI reduction were clearer in the fitted time series graphs (Figure 2). The HAI rate in the control group showed an abrupt and 1-time decrease at the end of the baseline period, from a baseline rate of 4.8 per 1000 patient-days to 2.8 (95% confidence interval [CI], 2.1 to 3.5), and did not change for the remaining 18 months of the study. The HAI rate in the intervention group declined from a baseline rate of 5.0 per 1000 patient-days in a gradual and exponential fashion throughout the intervention and follow-up periods to 2.1 per 1000 patient-days (95% CI, –0.4 to 4.5) without reaching a stable rate at the end of the study period, which might suggest continued intervention effects. The differences between the 2 study groups were not statistically significant. The rates of individual HAIs are shown in Table 2, and the differences in rates between intervention and control groups were not statistically significant. Of the 230 patients with HAI, 65 (28.3%) patients had 92 complications: 5 (5.4%) permanent loss of organ or organ system function, 55 (59.8%) transfers to higher level of care, and 32 (34.8%) deaths. The baseline rate of HAIs associated with complications was 1.57 and 0.87 per 1000 patient-days in the control and intervention groups, respectively. Both groups experienced a decrease in the rate of complications over time, to 0.47 and 0.53 per 1000 patient-days respectively, during the follow-up period (*P* = .03), but the difference between the groups over time was not statistically significant.

**Table 2.** **Rates of Individual Health Care–Associated Infections**

	Group	6-mo Baseline Period	9-mo Intervention Period	9-mo Follow-up Period
Rate of CLABSI	Control	0.84	0.49	0.48
	Intervention	0.98	0.74	0.4
Rate of CAUTI	Control	1.05	0.56	0.68
	Intervention	1.2	0.47	0.46
Rate of HAP	Control	1.36	1.05	1.16
	Intervention	0.87	0.74	0.99
Rate of CDI	Control	1.25	0.77	0.82
	Intervention	1.64	1.28	0.4

Differences in rates of individual HAIs between intervention and control groups were not statistically significant.

Abbreviations: CAUTI, catheter-associated urinary tract infection; CDI, *Clostridium difficile* infection; CLABSI, central line–associated bloodstream infection; HAI, health care–associated infection; HAP, hospital-acquired pneumonia; HCP, health care personnel.

## DISCUSSION

Our study gave us several insights into the implementation of positive deviance as a strategy to influence social behaviors among health care personnel and potentially change patient safety culture with the goal of influencing rates of health care–associated infections from the grassroots level in a busy academic medical center. Our paper adds to the methodology of implementing positive deviance intervention described in previous publications [22, 23]. The invitation to participate in PD was received well and generated a lot of conversation among the HCP in intervention wards. PD strategy, although labor intensive and “high touch,” allowed for increased engagement and awareness among frontline HCP. The culture of safety did not worsen among the HCP who received positive deviance intervention as it did among HCP in the control wards, and the difference between trends in the intervention and control wards was statistically significant. It is possible that open dialogues regarding challenges and potential solutions for preventing infections in the 3 intervention wards prevented a downward slide in the culture of safety, as was the case in the 3 control wards, during a time of organizational stress and aggressive system improvement that was being implemented at that time. This study was unable to determine whether the differences in culture of safety between the intervention and control wards were clinically impactful because the secular effects of systemwide hand hygiene efforts might have been a major contributor to reduction in HAI in both the intervention and control wards. There were no other significant events in the study wards that might have potentially explained the differences observed in patient safety culture between the groups. Any potential differences in patient complexity between the intervention and control wards and the study results were not studied. The variation in culture of safety between wards of similar size and the changes over time suggest that hospital culture of safety results need to be examined carefully at the level of each ward and that it might be helpful to measure them serially over time.

Network maps helped us identify connection patterns among HCP in the wards to prevent infections among the patients they care for. We found a concentration of attention in the patient’s nurse, patient care technician, charge nurse, ward manager, and ward clerk. This qualitative information was valuable. Communication intended to have a wide impact in any ward may be disseminated better through HCP with the highest number of relationships within the ward. Inclusion of social science experts in the multidisciplinary work groups for patient safety initiatives may be helpful.

Our study has some limitations. Interpretation of changes in culture of safety and the network survey is limited by the voluntary nature of participation in these surveys. The study was conducted at a single institution. Process measure data such as adherence to central line bundle and urinary catheter bundle were not available during the study period. Because physicians provide services throughout the hospital and rotate frequently, we were unable to include them in the intervention or the culture of safety surveys and network surveys in the study. The impact of PD on physicians’ perceptions of culture of safety is worth evaluating in future studies. Another limitation is that 9 months each for PD inquiry and follow-up may not have been sufficient in hindsight. We hope that the lessons learned in our study will be applied in future studies.

HAI rates decreased in both the intervention and control wards, indicating the effectiveness of systemwide aggressive hand hygiene measures. The average monthly hand hygiene observations in the entire hospital and health system increased 23-fold compared with the months before systems improvement agreement, and adherence increased from 93% to 98.5% (*P* < .05). Similarly, the environmental rounding observations increased 26-fold, and adherence increased from 91.9% to 92.8% (*P* < .05) (Parkland Infection Prevention Department, unpublished data 2013). The patterns of reduction were different, with gradual-exponential decline in the intervention wards, in contrast to 1-time, abrupt improvement in the control wards. We are unable to ascertain whether the differences in patterns could be explained by intervention effect.

Valuable insights we gained from conducting this study were:

1. It took time to gain the necessary trust from the frontline HCP in order for them to share freely their perceived barriers and potential solutions. Initiatives to reduce HAIs need to consciously factor in the time it takes to establish trust with participants in the intervention.
2. Once the HCP recognized the potential value of PD inquiry, they became engaged and shared many ideas with the study team. They found the conversation refreshing and made statements like, “I did not know my opinion mattered.” They also solved many seemingly small issues between themselves during the course of the conversation. For example, they came up with a color scheme to label intravenous tubing to identify which tubes needed to be changed on which day of the week. Staff modeled complex workflows to each other and learned from each other. Examples are workflows associated with drawing blood from a patient in contact isolation or cleaning up a patient with explosive diarrhea who has soiled himself (without contaminating catheter sites).
3. The ward managers of the intervention wards pointed to issues of time and the challenge of keeping a united front between different shifts. Local factors like these may be important for ongoing evaluation of infection prevention initiatives.
4. Although PD is a grassroots-level intervention, leadership buy-in and permission to engage with the research team were essential.
5. The research team and ward managers recognized that the PD approach is similar to the traditional quality improvement approach using Plan-Do-Study-Act cycles in that they have similar steps, but different in that the PD approach has an upfront phase of in-depth inquiry that is intended to get at the root of the problem, identify local role models for accelerating change, and emphasize local ownership of the problem and solutions.
6. Although the ward managers and frontline personnel eventually accepted PD after initial hesitation, the investigators identified a need for better tools to assess setting, context, and preconditions that might be more conducive for implementing PD. The approach also needs to be more developed and adapted for use in infection prevention because there is not a 1:1 correlation between any single practice by HCP and infection outcome in the patient. Another challenge in large, complex health systems is that peer role models are harder to identify and any single HCP may not have a deep reach within the area where they work.

In summary, when organizational conditions were the same for hospital wards that received positive deviance intervention and those that did not, PD inquiry of health care personnel was associated with a statistically significant impact on culture of safety. Mapping of social networks yielded several important insights about patterns of connections among frontline health care personnel. Rates of health care–associated infections and complications reduced in both intervention and control wards due systemwide interventions. Further studies are needed to evaluate social dynamics among health care personnel and their impact on culture of safety and HAI outcomes.

## Notes

***Financial support. ***This work was supported by the University of Texas System (Patient Safety Grant #137911; PI: Pranavi Sreeramoju, MD) and in-kind support provided by Parkland Health and Hospital System, Dallas, Texas.

***Potential conflicts of interest. ***All authors: no reported conflicts of interest. All authors have submitted the ICMJE Form for Disclosure of Potential Conflicts of Interest. Conflicts that the editors consider relevant to the content of the manuscript have been disclosed.
